# Simplification of Deep Neural Network-Based Object Detector for Real-Time Edge Computing

**DOI:** 10.3390/s23073777

**Published:** 2023-04-06

**Authors:** Kyoungtaek Choi, Seong Min Wi, Ho Gi Jung, Jae Kyu Suhr

**Affiliations:** 1 Department of AI Automation Robot, Daegu Catholic University, 13-13 Hayang-ro, Hayang-eup, Gyeongsan-si 38430, Gyeongsangbuk-do, Republic of Korea; 2 Driving Image Recognition Logic Cell, Hyundai Mobis, 17-2 Mabuk-ro 240beon-gil, Giheung-gu, Yongin-si 16891, Gyeonggi-do, Republic of Korea; 3 Department of Electronic Engineering, Korea National University of Transportation, 50 Daehak-ro, Chungju-si 27469, Chungbuk-do, Republic of Korea; 4 Department of Intelligent Mechatronics Engineering, Sejong University, 209 Neungdong-ro, Gwangjin-gu, Seoul 05006, Republic of Korea

**Keywords:** object detector, network simplification, channel pruning, edge computing

## Abstract

This paper presents a method for simplifying and quantizing a deep neural network (DNN)-based object detector to embed it into a real-time edge device. For network simplification, this paper compares five methods for applying channel pruning to a residual block because special care must be taken regarding the number of channels when summing two feature maps. Based on the comparison in terms of detection performance, parameter number, computational complexity, and processing time, this paper discovers the most satisfying method on the edge device. For network quantization, this paper compares post-training quantization (PTQ) and quantization-aware training (QAT) using two datasets with different detection difficulties. This comparison shows that both approaches are recommended in the case of the easy-to-detect dataset, but QAT is preferable in the case of the difficult-to-detect dataset. Through experiments, this paper shows that the proposed method can effectively embed the DNN-based object detector into an edge device equipped with Qualcomm’s QCS605 System-on-Chip (SoC), while achieving a real-time operation with more than 10 frames per second.

## 1. Introduction

Object visual detection, which estimates the position and type of an object, is an essential computer vision technology and has been used in various fields such as surveillance systems, autonomous driving, robots, and smart factories. Because object detection has a wide range of applications, it must operate on a variety of devices, from high-performance servers to edge computing devices such as surveillance cameras, mobile phones, self-driving cars, and micro-drones. Currently, most object detectors are based on deep neural networks (DNNs), and although they show excellent detection performance, they require a large amount of computation. Achieving both high detection performance and real-time processing is a very important issue in time-critical applications such as autonomous driving, missiles, and smart factories. Moreover, these time-critical applications should often operate on an edge device whose computing power is limited. To port these applications on an edge device, the network compression that optimizes the neural network to satisfy both the time constraints and the performance requirements is essential [1,2,3,4,5]. Network compression consists of network simplification, which simplifies the network architecture, and parameter quantization, which compresses the bit width of parameters to lower than floating point. For network simplification, there are tensor decomposition [6], knowledge distillation [7], neural architecture search (NAS) [8], and network pruning [9]. Among them, network pruning is very popular because it is effective in easily reducing the computational load and memory while maintaining detection performance [3]. The combination of network pruning and parameter quantization is the most popular method for network compression [10]. Network pruning, like other network simplification methods except NAS, requires the original neural network to have the qualified performance. Fortunately, object detection, an application field covered in this paper, has been studied for a long time and there are many open-source detectors with excellent performance [11,12]. Therefore, we selected one of the most popular DNN-based detectors, YOLOv4 [13]. YOLOv4 has been proven for a considerable period in various applications [14,15] and shows a compromise between computational cost and detection accuracy in various frameworks [16,17]. We have ported this detector onto Qualcomm’s QCS605 [18] using pruning and parameter quantization. In this paper, we analyze commonly used pruning methods and representative quantization methods through experiments and provide guidelines for porting deep neural networks to edge devices based on the results. The contributions of this paper are as follows. This paper presents a method for embedding a DNN-based object detector onto an edge device using network pruning and parameter quantization. It also demonstrates the real-time performance of this method in a traffic surveillance camera equipped with the QCS605. In particular, this paper proposes the best method for pruning neural networks that include residual blocks [19] through comparative studies. Additionally, the paper finds that there can be a significant performance gap between two representative quantization methods, post-training quantization (PTQ) [20] and quantization-aware training (QAT) [21], depending on the difficulty of the dataset. Some issues to consider for porting DNNs in edge devices are also covered, and these issues are analyzed through various experiments.

The rest of this paper is organized as follows. Section 2 introduces the work related to network compression. Section 3 explains the methods used in the comparative studies of network compression. Section 4 presents the experimental results and analyses. Finally, this paper concludes in Section 5.

## 2. Related Research

To reduce the computations and memory of a DNN, various network simplification methods have been studied [1,2,3,4,5]. Network simplification methods can be categorized into tensor decomposition, network pruning, knowledge distillation, and NAS. Tensor decomposition and network pruning focus on simplifying highly accurate, but complex networks. Knowledge distillations focus on transferring the knowledge from a complex network to its simplified version to mimic the complex one. NAS focuses on automatically generating an optimal network by combining network primitives in the search space.

Tensor decomposition can be divided into low rank matrix decomposition methods and tensorized decomposition methods [1]. Low rank matrix decomposition methods are mainly based on singular value decomposition (SVD) and decompose a weight matrix into the product of multiple low rank matrices [22]. Yang et al. proposed SVD training that adds the regularized term of weight matrices to the original loss to decrease the ranks of the weight matrices [23]. Chen et al. proposed joint matrix decomposition that simplifies multiple layers with the same structure simultaneously [24]. Tensorized decomposition methods substitute a high-dimensional tensor with the product of multiple low-dimensional tensors. These are Tucker decomposition (TD), canonical polyadic decomposition (CPD), tensor train (TT), and tensor ring (TR) in the tensor decomposition [25,26,27,28]. In tensor decomposition, the lower the rank of decomposed tensors, the lower the amount of computation, but the lower the accuracy of the network. Therefore, how to reduce the rank of tensors as much as possible while maintaining network accuracy and how to train the decomposed network to avoid accuracy degradation are important issues. Kim et al. suggested a tensor decomposition method that selects the rank of tensors to be decomposed based on the variational Bayesian matrix factorization and decomposes a tensor using TD [25]. Phan et al. suggested a method to avoid the degeneracy problem caused by tensor decomposition, and their method is based on CPD [26]. Yin et al. proposed a tensor decomposition framework that consists of a regularized training procedure, tensor decomposition, and fine tuning [27]. The regularized training procedure trains the uncompressed network to increase its accuracy while gradually reducing the tensor rank of the network. This is similar to the sparsity training of network pruning. Tensor decomposition can considerably reduce the amount of computation and memory, but it may not significantly reduce the inference time. Tensor decomposition substitutes a high-order tensor with the product of multiple low rank tensors, which means that a single layer is converted to a sequence of layers. This makes the parallel processing of a network more difficult, because in a sequence of layers, a layer should wait for the output tensor of its previous layer.

Network pruning can be categorized into an unstructured method and a structured method, or categorized into a static method and a dynamic method [29]. Unstructured pruning means to remove each weight of a filter in a network individually [30,31,32]. Because of the individual removal of the weights, a dedicated operation library or hardware is required in order not to perform operations on the removed weights. There is also no change in the capacity of the feature map before and after pruning, so there is little memory compression of the network. Structured pruning does not remove each weight of a filter, but rather a structured element of a network, such as a channel or a layer [33,34,35,36,37], and it can reduce both memory consumption and computation without any specific hardware or software. In the beginning, there was a method of removing neurons whose activation output was very close to 0 regardless of their input [37] or removing a channel with low importance estimated by LASSO regression [33,34]. Following this, Liu et al. proposed a network slimming method that estimates channel importance with a scaling factor in batch normalization [35]. Yu et al. proposed a neuron importance score propagation (NISP) method that estimates the contribution of each channel to the final outputs of a network [38]. Zhuang et al. proposed a discrimination-aware channel pruning (DCP) method to estimate channel importance by adding a discrimination-aware loss into the intermediate layers of a network [36]. Structured pruning, unlike unstructured pruning, requires no dedicated software or hardware. However, since it removes all weights belonging to the same element, it can degrade the performance compared to unstructured pruning.

Knowledge distillation (KD) involves training the simplified network to make its output similar to the output of the original network [7,39]. In general, teacher networks and student networks have the same final output structure, but the internal structure of the networks is different, so knowledge is only transferred through the final output. Li et al. proposed a method that makes the internal output structure the same between the student network and the teacher network by attaching a 1*1 ad hoc convolution layer to the output of the layers or blocks [40]. Yang et al. proposed a knowledge distillation-based method to train the student network to work well in the target domain with unlabeled data alone [41]. This method creates a similar final output distribution in the target domain between a teacher network and a student network by adding the Kullback–Leibler (KL) divergence to the conventional knowledge distillation loss. Duong et al. proposed a method to minimize angular distillation loss instead of KL divergence to make the final output distributions similar [42]. This loss is similar to the cosine distance widely used in face recognition. The structures between a teacher network and a student network are generally different. However, in order to solve the network overfitting, Yun et al. proposed the self-knowledge distillation method, whose teacher and student network are the same [43]. This method trains a network to have a similar output distribution for two different input vectors with the same labels. As mentioned, the main purpose of knowledge distillation is not to simplify the network, but to increase the performance of the student network by utilizing the teacher network. In knowledge distillation, the student network can be generated independently from the teacher network, but is mainly generated from the teacher network by tensor decomposition or network pruning.

Neural architecture search (NAS) is used to generate the network architecture by searching the predefined space, evaluating the architecture, and repeating these procedures until the optimal network is found [8,44]. NAS, unlike tensor decomposition and network pruning, has the advantage of finding the optimal network that is not bounded by the original network. However, NAS generally takes too much time to find the optimal network because of the huge searching space. Therefore, how to define the searching space, how quickly to find a network candidate in that space, and how to evaluate the candidate are key issues for NAS. Generally, DNN consists of the repetition of subgraphs. Therefore, NAS defines the primitives of the searching space as small subgraphs such as convolutional layer or residual block, and designs the whole network by combining these subgraphs [45,46,47]. NASNet finds the best convolutional layer with a small database and evaluates all network candidates consisting of the identified layers with a large database [45]. MnasNet presents a factorized hierarchical search space that factorizes a CNN model into unique blocks consisting of multiple layers, and parameters related to a layer. This method also evaluates the network candidates by considering the inference latency on an edge device [48]. For searching algorithms in NAS, there are random search [49], Bayesian optimization [50], reinforcement learning (RL) [8,45], and neuroevolution [51,52,53]. It is difficult to conclude which algorithm is best, but there are some references that include comparison results [54,55]. The naïve methods used to fully train model candidates and evaluate them are out of date. More effective methods to predict the accuracy of model candidates have been studied [56], and one-shot NAS only trains one supernet, subsuming all model candidates, and each model is cheaply evaluated by inheriting its weights from the supernet [57].

Typically, DNNs in conventional computers store and perform operations on model parameters in floating-point format. However, most edge devices have accelerators dedicated to integer operations only. Therefore, quantization is performed to convert 16-bit or 32-bit floating-point numbers into 8-bit integers. Some networks even convert floating-point operations into binary operations in extreme ways, but in such cases, separate hardware development is required to support these networks [58]. Parameter quantization can be divided into post-training quantization (PTQ), which quantizes parameters after network training, and quantization-aware training (QAT), which trains the network while considering quantization [21]. Post-quantization can also be divided into two groups, one requiring the trained network only and the other requiring additional input data [20]. These quantization methods are easy to apply because they are implemented in a public software library [59].

The method of optimizing DNNs through pruning and quantization has been widely used. However, there is a problem regarding the pruning of networks that include residual blocks. While there are a few solutions to this problem, no literature compares and analyzes them to suggest the best method. In addition, there is a lack of literature that compares and analyzes the performance difference between the two representative quantization methods, PTQ and QAT, according to the difficulty of the dataset. Therefore, this paper suggests several methods for pruning residual blocks and compares them with existing methods through experiments to find the optimal approach. This paper also presents experimental comparisons between PTQ and QAT according to the difficulty of the dataset.

## 3. Comparative Studies for Network Compression

### 3.1. Network Architecture and Overview of the Porting Process

**Figure 1:**
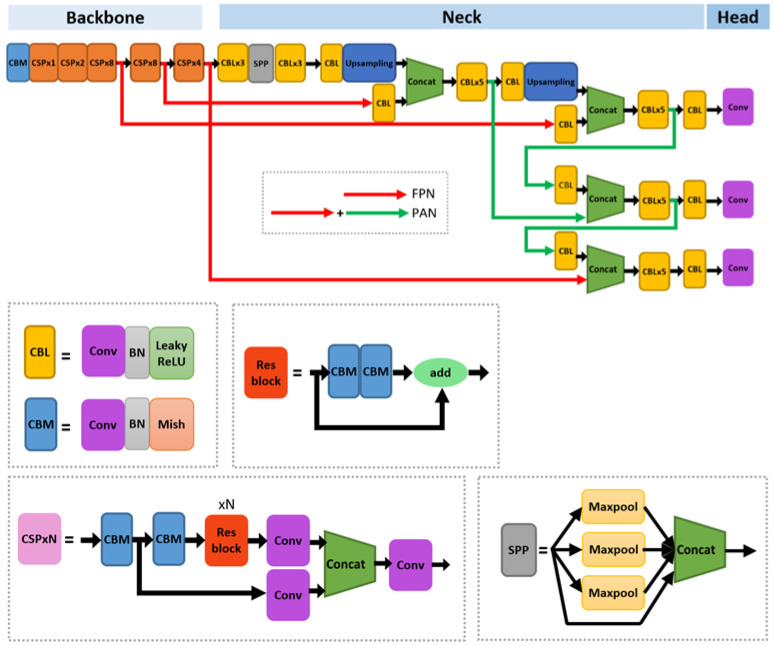
Network architecture of YOLOv4.

This paper deals with neural network compression methods for porting DNN-based object detectors to edge devices. The object detector used in this paper is YOLOv4 [13]. The YOLO-series detector is a one-stage detector that simultaneously estimates the position and the class of an object. One-stage detectors are known to be faster than two-stage detectors that estimate the position first and then the class [11]. The architecture of YOLOv4 is shown in Figure 1. The basic convolution blocks in YOLOv4 consist of convolution, batch normalization and Mish activation or convolution, batch normalization, and leaky ReLU activation. These convolution blocks are denoted as CBM or CBL in Figure 1. When porting YOLOv4 into the edge device equipped with Qualcomm QCS605, Mish activation [60] was substituted with leaky-ReLU [61], since Mish activation is not supported by Qualcomm’s library. As shown in Figure 1, YOLOv4 consists of a backbone for extracting general features, a neck for extracting multiscale detection features, and a head for outputting the final results. The backbone adopts a CSP (cross-stage partial connections) block [62] that splits the input channels before dense connections to reduce memory and computation, and a CSP block contains a residual block, as shown in Figure 1. CSPx4 in Figure 1 denotes that the CSP block appears four times in a row. In the neck of YOLOv4, SPP denotes a Spatial Pyramid Pooling block to combine low- and high-resolution features [63]. Finally, Concat and Conv in Figure 1 denote concatenation and convolution, respectively.

**Figure 2:**
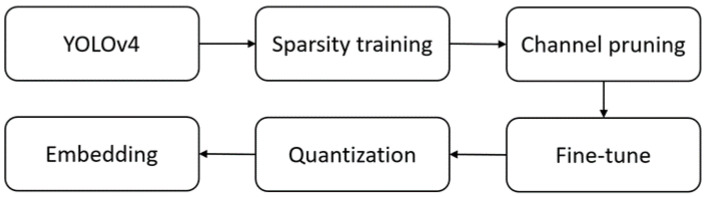
Process to port YOLOv4 to an edge device.

The process to port the object detector to an edge device is shown in Figure 2. First, for the network compression, sparsity training is performed to permit a network to transfer major information through only a small number of channels in each layer. Then, less important channels that do not transfer much information are pruned, and the pruned network is retrained to restore its performance in the fine-tuning phase. After the fine-tuning phase, the network parameters are quantized and the network is finally ported to an edge device. These final phases may vary depending on the quantization method and device type.

### 3.2. Simplification

The number of channels of the output tensor of a convolutional layer is equal to the number of filters, so the amount of computation and memory are proportional to the number of channels of the output tensor. There may be relatively fewer informative ones among the channels of the output tensor. By pruning these less informative channels, the amount of computation and memory can be reduced, while maintaining the network accuracy.

Generally, in a convolutional neural network, a convolutional layer is followed by a batch normalization layer. The batch normalization layer uses statistics from the training data to normalize the output tensor of the convolutional layer, as shown in Equation (1), and then applies a scale factor γ and bias β to the normalized tensor, as shown in Equation (2) [64].

(1) $$x_{norm}=\frac{x-\mu }{\sqrt{\sigma^{2}+\epsilon }}$$

(2) $$y=\gamma \cdot x_{norm}+\beta$$

γ and β are trainable parameters, and a low absolute value of trained γ of a channel means that the corresponding channel transfers little information to the next layer; that is, the channel may be not informative. A training method that reduces the original cost of a neural network and the L1 norm of γ at the same time is called sparsity training [9,65]. After sparsity training, most of the information is transferred through a few channels, so lots of channels become unnecessary. The cost function of sparsity training is to weight the sum of the L1 norms of γ by α and then add it to the original cost of the network, as shown in Equation (3) [66].

(3) $$\mathrm{Loss}=loss_{original}+\alpha \underset{\gamma =\Gamma }{\sum }\|\gamma \|$$

After sparsity training, the channels with γ below the threshold are pruned and the performance degraded by pruning is restored by fine tuning, as shown in Figure 2.

**Figure 3:**
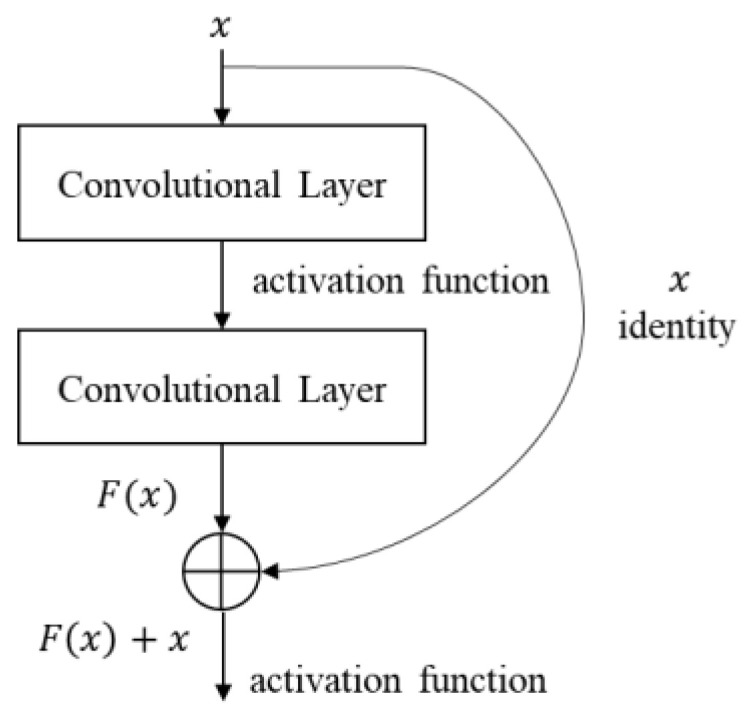
Residual block structure in Resnet.

**Figure 4:**
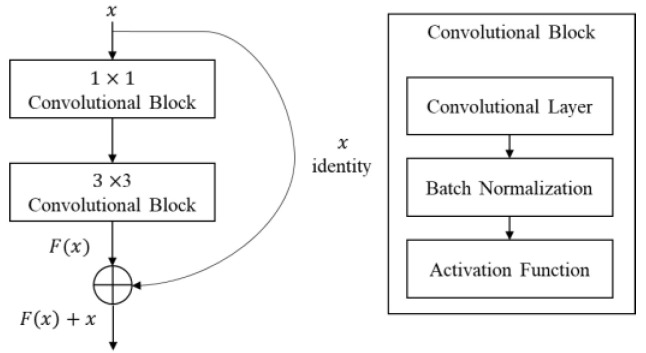
Residual block structure in YOLOv4.

When pruning a convolutional neural network, residual blocks with a shortcut (skip connection) need a special care [19]. A residual block is a network architecture that adds a detour shortcut of two convolutional layers to avoid vanishing or exploding gradients, as shown in Figure 3. The output tensor of a residual block is the sum of the block’s input tensor and the output tensor of the convolutional layers within the block, as shown in Figure 3; Figure 4. To calculate the sum of these two tensors, the channel number and channel indices of these tensors must be the same. If the channels of a tensor in a residual block are pruned individually, the channel number and channel indices of a tensor become different and the sum of two tensors cannot be correctly calculated. This paper describes five methods for pruning residual blocks in the following subsections and compares their performances in an edge device.

#### 3.2.1. Skip Method

**Figure 5:**
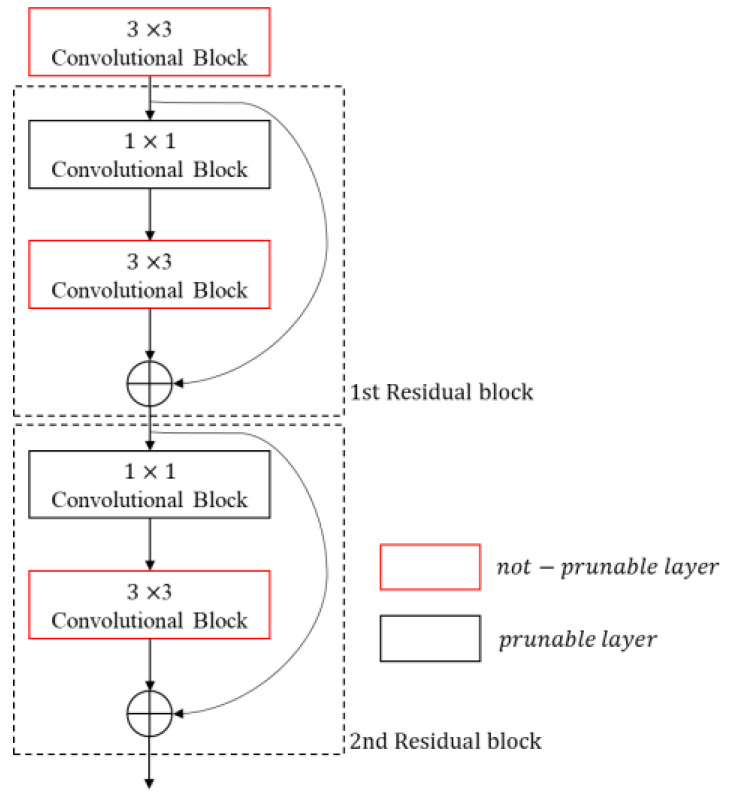
Skip method.

The skip method only prunes the remaining layers except for the 3 × 3 convolutional layers bypassed by shortcut in a residual block, as shown in Figure 5. Although this method is simple to implement, it has the limitation that it cannot prune the computationally heavy 3 × 3 convolutional layer.

#### 3.2.2. Head-First Method

**Figure 6:**
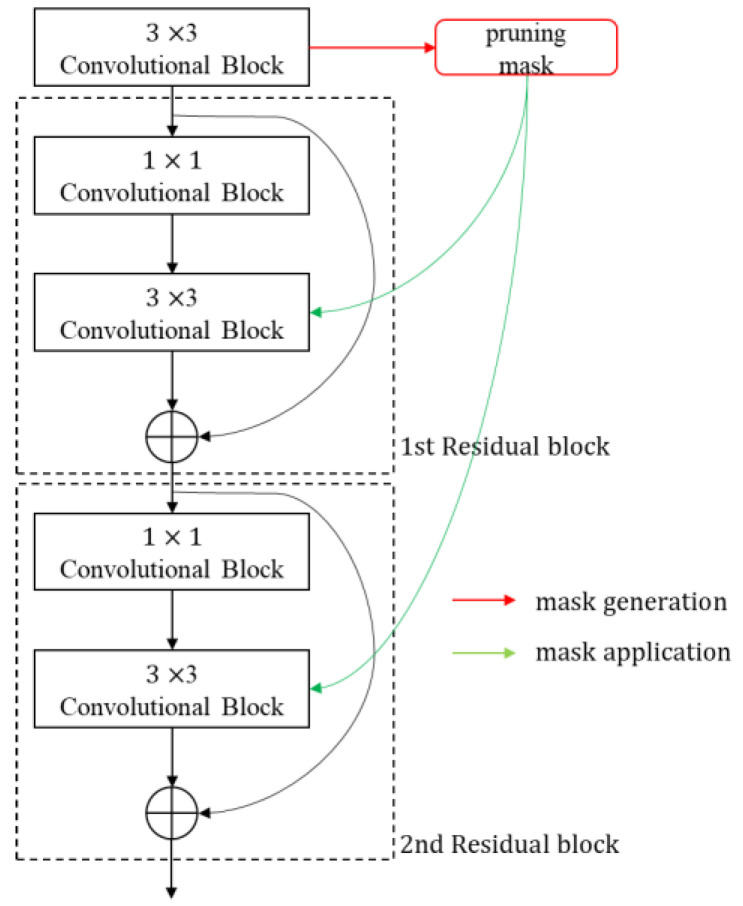
Head-first method.

The head-first method was proposed by Li et al. [67]. The head-first method generates the pruning mask from the first 3 × 3 convolutional layer in the sequential residual blocks as shown in Figure 6 and applies the same mask to the other 3 × 3 convolutional layers connected by shortcuts. As illustrated in Figure 6, when residual blocks are sequentially connected, the information of the output tensor of the first 3 × 3 convolutional layer is passed to the end of the blocks through shortcuts, so the pruning mask of this tensor is applied directly to the output tensor of other 3 × 3 convolutional layers. This method is also easy to implement and, unlike the skip method, the pruning of the 3 × 3 convolutional layer is possible, but some of the important channels of the 3 × 3 convolutional layer can be pruned, which may cause performance degradation.

#### 3.2.3. OR Method

**Figure 7:**
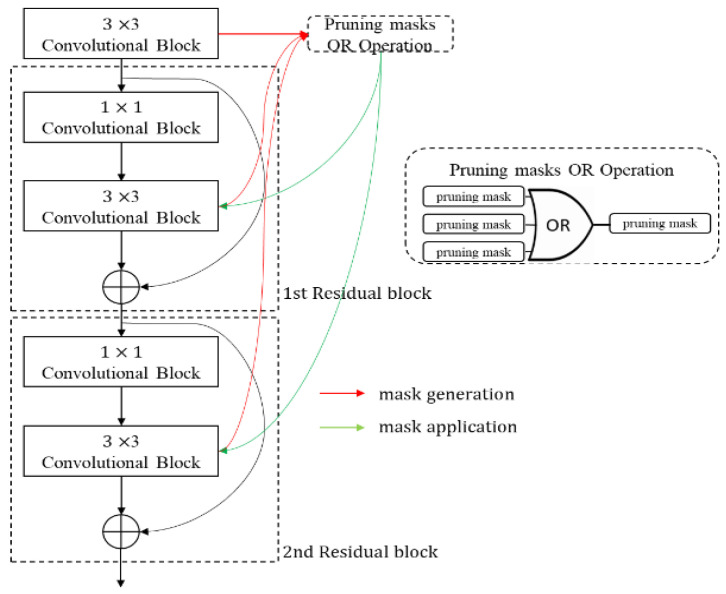
OR method.

The OR method only prunes those channels that are prunable in all 3 × 3 convolutional layers within residual blocks and is introduced in SlimYOLOv3 [66]. As shown in Figure 7, the OR method generates a new mask by the logical OR operation of the pruning masks of each 3 × 3 convolutional layer within residual blocks and applies the new mask to all 3 × 3 convolutional layers. Unlike the head-first method, this method has no risk of pruning important channels, but conversely, it also cannot prune unimportant channels.

#### 3.2.4. Slice and Concatenation Method

**Figure 8:**
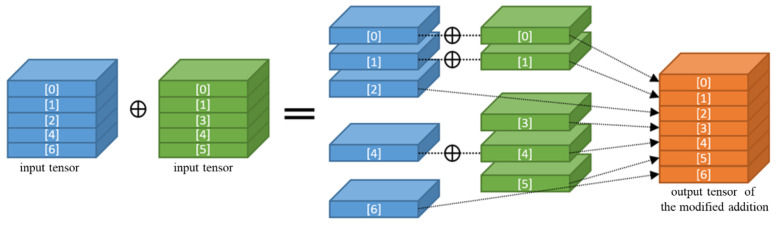
Slice and concatenation.

This method modifies the addition operation in a residual block instead of the pruning mask. This method prunes the channels of each convolutional layer individually, and the modified addition operation merges two channels with the same index by addition and concatenates unpaired channels to the output, as shown in Figure 8. This method slices the input tensors of the addition operation into channels and processes each channel individually using loop and conditional statements. Processing channels individually generates many nodes in Tensorflow’s graph [68] and it dramatically increases the inference times on both edge devices and regular computers with high-performance GPUs. For this reason, this method was excluded from the comparative experiments.

#### 3.2.5. Gather Method

**Figure 9:**
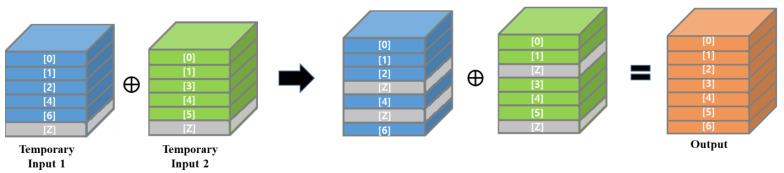
Gather method.

The gather method also prunes convolutional layers individually and modifies the addition operation in a residual block. In order to make the channel number and channel indices of the two input tensors of the addition operation the same, this method pairs the unpaired channels with all zero-valued channels and then adds two input tensors together, as shown in Figure 9. Unlike slice and concatenation, this method performs the addition between the tensors rather than the channels, thus reducing the generation of operational nodes. We implemented this method using Tensorflow’s gather function [69] that gathers the channels selected by the list of channel index. For example, this method creates temporary input tensors by concatenating a zero-valued channel (denoted [Z] in Figure 9) to the pruned input tensors. Then, as shown in Figure 9, if the original channel indices of the two pruned input tensors are [0,1,2,4,6] and [0,1,3,4,5], in order to make the two input tensors have the same dimension and channel indices, this method extracts the [0,1,2,Z,4,Z,6] channels and [0,1,Z,3,4,5,Z] channels, respectively, from two temporary input tensors using the gather function. This method achieves the same pruning result as the slice and concatenation method, while minimizing the operational node generation.

#### 3.2.6. Concatenation and Convolution Method

**Figure 10:**
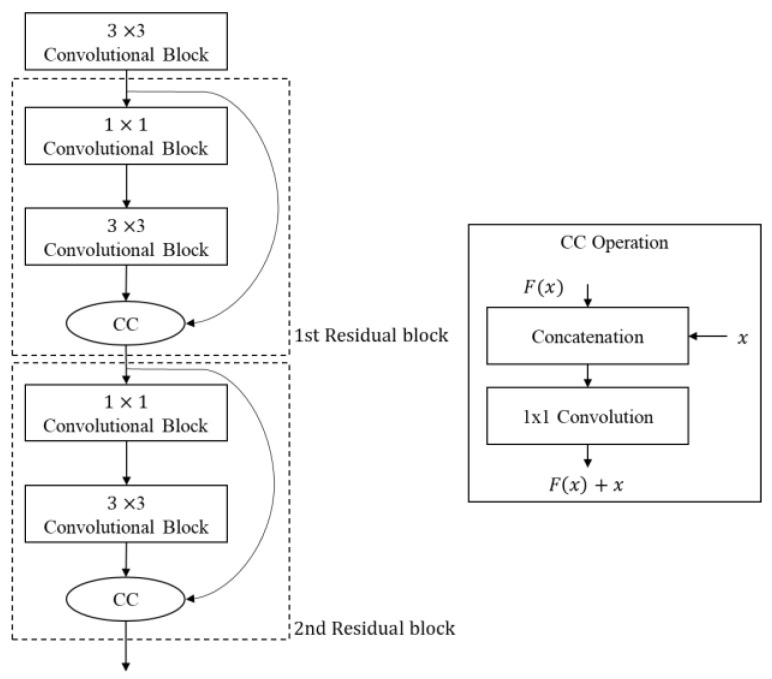
Concatenation–convolution.

The process of slicing a specific channel from a tensor or merging multiple channels into one can be implemented with 1 × 1 binary filter convolution. In order to prune the channels of each layer individually, the addition in a residual block can be modified as concatenation–convolution (CC) operation by using 1 × 1 convolution, as shown in Figure 10. First, the two pruned input tensors of the CC operation are concatenated along the channel axis. Then, via 1 × 1 convolution, the unpruned channel pairs from both tensors are merged into one, and otherwise unpaired channels are simply extracted and concatenated. For this, the filter coefficients of the 1 × 1 convolution are generated from the pruning masks of two input tensors. As this method allows the individual pruning of channels in all layers based on their importance, it can effectively simplify a neural network while maintaining high network performance. However, the computations for the 1 × 1 convolution in CC operation is not ignorable.

### 3.3. Batch Normalization Folding and Quantization

In addition to the network simplification mentioned above, porting a network to an edge device requires the parameter quantization and the folding process that combines a convolution with a batch normalization. In general, a convolution block consists of convolutional, batch normalization, and activation layers. After all network parameters (filter coefficients, mean, variance, scale factor γ and bias β of channels, etc.) have been trained, the convolutional and batch normalization layers can be combined as in Equation (8) to reduce computations. This is so-called batch normalization folding [20].

(4) $$y_{conv}=W_{conv}\cdot x+b_{conv}$$

(5) $$y_{BN}=\gamma \frac{y_{conv}-\mu }{\sqrt{\delta^{2}+\epsilon }}+\beta$$

(6) $$W_{fold}=\gamma \frac{W_{conv}}{\sqrt{\delta^{2}+\epsilon }}$$

(7) $$b_{fold}=\gamma \frac{b_{conv}-\mu }{\sqrt{\delta^{2}+\epsilon }}+\beta$$

(8) $$y_{BN}=W_{fold}\cdot x+b_{fold}$$

**Figure 11:**
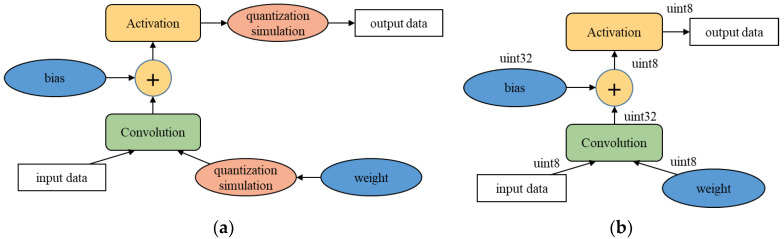
Quantization aware training. (**a**) Network architecture for the simulation of the quantized network; (**b**) quantized network after QAT.

Equations (4) and (5) are for convolution and batch normalization, respectively. Since both convolution and batch normalization are linear transformations, the weight and bias of the convolution are combined with the parameters of batch normalization, as in Equations (6) and (7), to calculate the weight and bias of the folding layer, respectively. After network simplification and batch normalization folding, parameter quantization is performed to port a network to an edge device that supports fast parallel integer multiplication. In this paper, the quantization method of Qualcomm’s software library was applied [59]. We compared post-training quantization and quantization-aware training. Post-training quantization (PTQ) quantizes network parameters after network training. This is easy to apply, but the quantization of the trained weights can lead to information loss, which can degrade the network performance. In particular, this network performance degradation easily occurs in binary or shallow neural networks [29]. PTQ is further subdivided into methods with or without input data. In this paper, PTQ, which does not require input data, is used for parameter quantization [59]. Unlike PTQ, quantization-aware training (QAT) trains neural networks by considering parameter quantization. To do this, the QAT method performs training by inserting auxiliary nodes to simulate the quantized version of an original network, as shown in Figure 11a. It is impossible to directly train the quantized network because there is a limit of precision in calculating the gradient of the loss and using it to update the network parameters. Therefore, instead of using quantized parameters, this method inserts simulation nodes to estimate the loss of the quantized version of the original network and trains the network to minimize the loss. After training, the network is quantized as shown in Figure 11b. In general, QAT shows better quantization performance than PTQ because this method trains the original network to guarantee the performance of the quantized version through simulation as much as possible. In this paper, the QAT suggested by Jacob et al. is applied to finely train the pruned network [21].

## 4. Experimental Results

**Figure 12:**
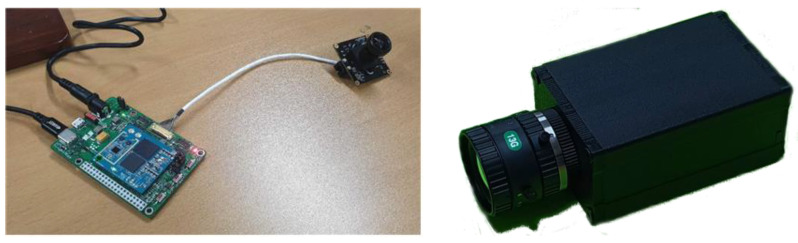
Intelligent camera equipped with Qualcomm QCS605.

To compare the aforementioned pruning methods and quantization methods, we developed an intelligent camera equipped with Qualcomm’s QCS605 (Figure 12). QCS605 is a System-on-Chip (SoC) that integrates CPU, GPU, and DSP for high-performance Internet of Things. It can be considered a small, low-power AI edge device because its dimensions are 78 × 52 × 38 mm, giving 2.1 TOPS at 1 watt. As the main applications of this camera, we considered visual surveillance and drones.

The process to port a deep neural network to Qualcomm’s chip is as follows. First, a neural network is created under a deep learning framework such as Tensorflow, Caffe, or ONNX. Then, the network is converted into a deep learning container (DLC) file using Qualcomm’s software development kit (SDK) called snapdragon neural processing engine (SNPE) [59]. The DLC file is ported to a device. In this paper, we ported YOLOv4 object detector into QCS605. Since SNPE does not support Mish and nearest neighbor interpolation used in YOLOv4, Mish and nearest neighbor interpolation were replaced by leaky ReLU and bilinear interpolation, respectively.

**Figure 13:**
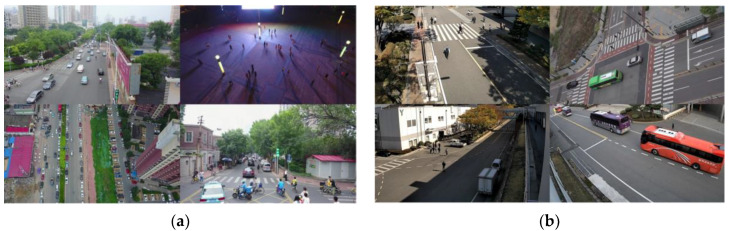
Experimental data sets. (**a**) Visdrone2019-Det; (**b**) SCOD.

For the experiment, we used a public dataset called VisDrone-DET2019 [70] collected from drones and a private dataset collected from our developed camera. The private dataset was named SCOD. In the case of VisDrone-DET2019, the detection difficulty is very high because most objects look small and their types vary, as shown in Figure 13a. In the case of SCOD, the detection difficulty is lower than VisDrone-DET2019 because most objects look relatively large and there are only three object types, as shown in Figure 13b. Table 1; Table 2 show the number of training and test objects according to object type in the VisDrone-DET2019 and SCOD datasets, respectively. For all experimental results except Section 4.4, the input images for the object detector were resized to 416×416×3 and 416×256×3 for the VisDrone-DET2019 and SCOD datasets, respectively.

### 4.1. Experimental Results for Quantization Methods

First, the mean average precision (mAP) according to the quantization methods for each dataset was compared, as shown in Table 3. In the SCOD dataset with relatively low detection difficulty, the performance difference between the non-quantized and quantized networks was small. However, in the case of VisDrone-DET2019 with high detection difficulty, QAT showed little performance degradation, but PTQ showed significant performance degradation compared to the non-quantized result.

### 4.2. Experimental Results for Sparsity Training

**Figure 14:**
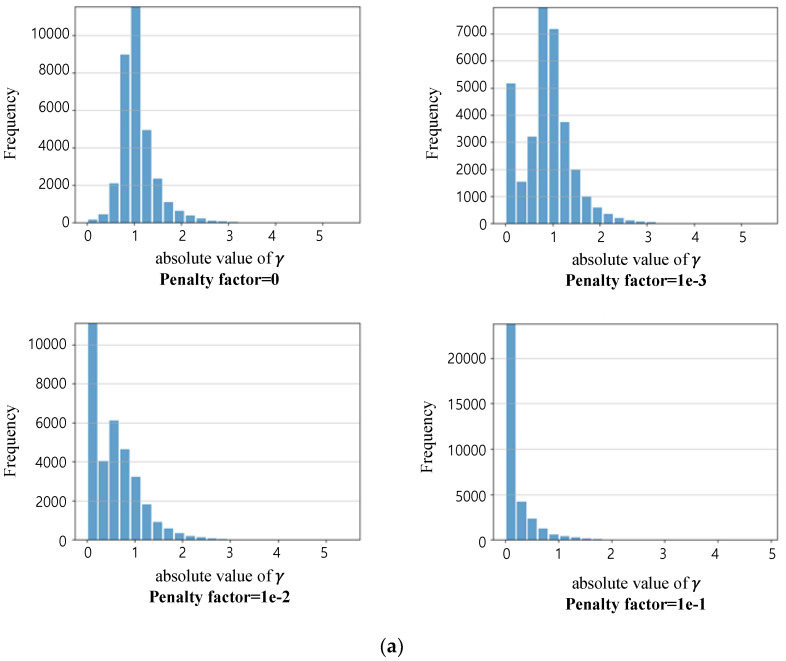

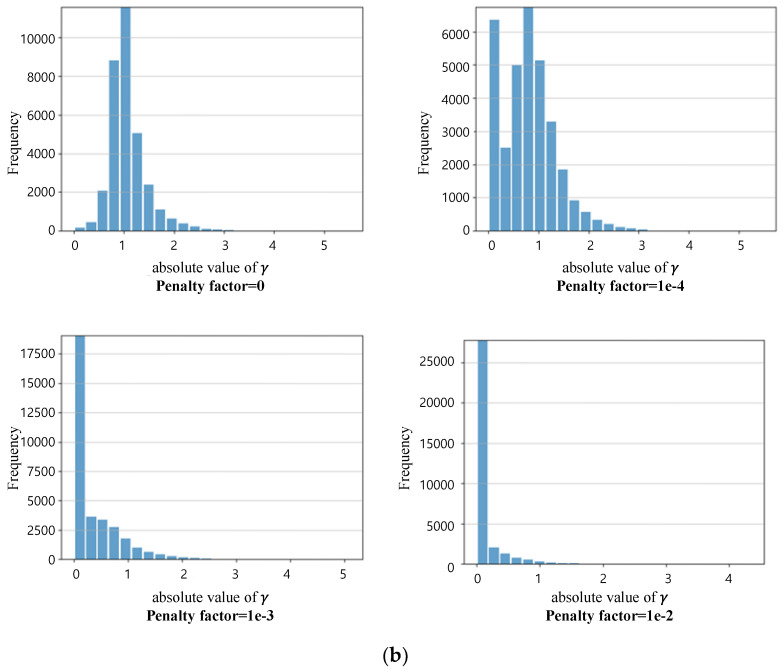
Histogram of γ according to the penalty factor α. (**a**) Visdrone2019-Det; (**b**) SCOD.

In order to prune a network, the network should be trained to minimize the loss, as shown in Equation (3). According to the penalty factor α in Equation (3), there is a trade-off between the detection performance and the information concentration on several channels in a network. Figure 14a,b shows the histograms of the scale factor γ of the network channels according to the penalty factor α for VisDrone-DET2019 and SCOD DB, respectively. As the penalty factor α increases, the number of channels with low γ, which indicates the importance of the channel, increases. That is, the number of channels that greatly contribute to the detection performance becomes small. Therefore, even if a significant number of channels are pruned, the performance degradation due to this is insignificant. However, if the penalty factor becomes too large, the original loss that the network is actually trying to minimize is less minimized and causes performance degradation. Table 4 shows the mAP according to the penalty coefficient after finishing sparsity training only. Based on Table 4 and Figure 14, we set the penalty factor $10^{-2}$ and $10^{-3}$ for VisDrone-DET2019 and SCOD, respectively, by considering the detection performance and the γ histogram according to the penalty factor.

### 4.3. Comparison of Pruning Methods Considering Residual Network Structure

YOLOv4 was pruned by applying five channel pruning methods considering the residual network structure, and the performances according to the pruning methods were compared in the server equipped with high-performance GPU and in QCS605. Table 5; Table 6 show the performances of the networks with channels 0%, 50%, and 70% pruned by the five pruning methods in VisDrone-DET2019 and SCOD, respectively. In Table 5; Table 6, all items except the inference time were measured only on the DSP of QCS605. The inference time was measured on both of the servers’ high-performance GPU (Nvidia Titan RTX 24GB) and QCS605′s DSP. The second column of Table 5; Table 6 denote the pruning methods. In the second column, the pruning methods SKIP, Head-First, OR, Gather, and Concatenation–Convolution were denoted by SK, HF, OR, GA, and CC, respectively. As shown in Table 5; Table 6, since the GPU has powerful computing power, the inference time according to the pruning ratio is not significantly different in the GPU. As shown in Table 5; Table 6, when applying the GA method in GPU, the inference time is similar to other methods, but in DSP, the time is nearly double that of other methods. We think that this occurs because Tensorflow’s gather function is not optimized for QCS605. The inference time of CC method is 10% longer than that of the SK, HF, and OR methods. This is because the amount of computation is increased by concatenation and 1 × 1 convolutional layer in CC operation. As shown in Table 5, the memory consumption and inference time of HF are less than others, but the detection performance is lowered by 1~2% compared to the SK or OR method as the pruning rate is increased. As shown in Table 6, the detection performance of the SK method is less than that of the others in the SCOD dataset whose detection difficulty is low. Considering the detection performance for each DB, memory consumption, and inference time, the OR method was found to be the best among the five methods.

**Figure 15:**
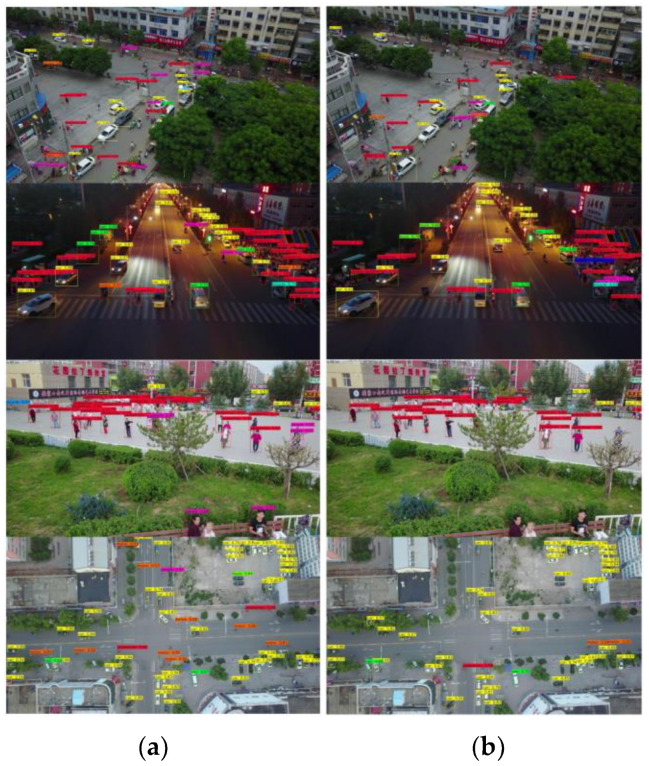
Detection results in Visdrone2019-Det. (**a**) Original version of YOLOv4; (**b**) YOLOv4 optimized for an edge device.

**Figure 16:**
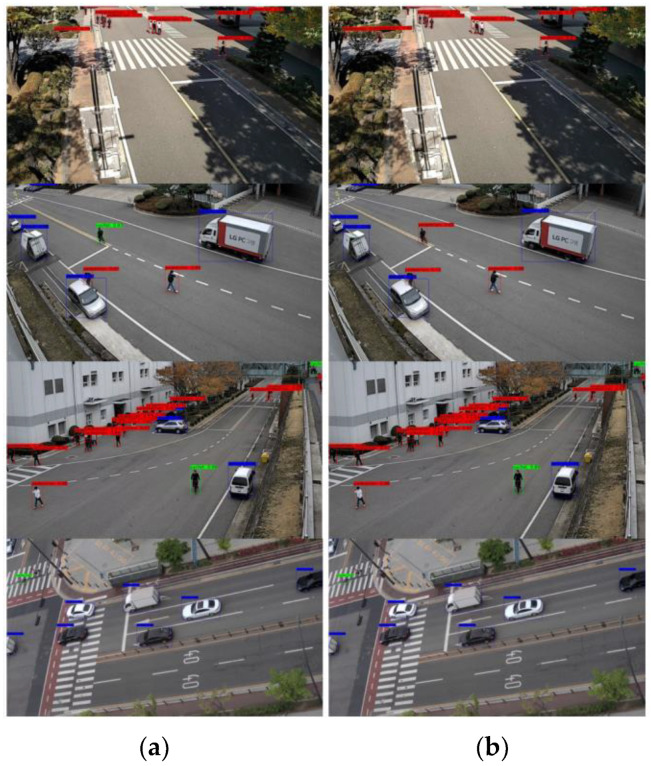
Detection results in SCOD. (**a**) Original version of YOLOv4; (**b**) YOLOv4 optimized for an edge device.

Figure 15; Figure 16 show the detection results in VisDrone-DET2019 and SCOD, respectively. In Figure 15; Figure 16, the left pictures show the detection results of the original YOLOv4 and the right ones show the detection results of the YOLOv4 ported to QCS605 through the simplification and the quantization. For the simplification of YOLOv4, 70% of the channels were pruned using the OR method. Although the pruned network missed a few small objects in VisDrone-DET2019, the detection performance of the pruned network was very close to that of the original, even though 70% of the channels were pruned.

### 4.4. Detection Performance according to Input Image Size and Pruning Ratio

The input image resolution to the network has a significant impact on the object detection performance. The lower the resolution of the input image, the shorter the inference time. However, if the detection objects are visually small, such as VisDrone-DET2019, the detection performance may be significantly degraded. In this paper, we developed an intelligent camera equipped with QCS605 that detects objects within 100 ms per image. To maximize the detection performance while satisfying the time constraint, the detector was evaluated with two datasets while adjusting the pruning rate and the input image resolution, as shown in Table 7. For pruning, the OR method was applied in the same way as in the previous experiment. As shown in Table 7, when the object is visually small, it is appropriate to increase the resolution of the input image for maintaining the detection performance and to increase the pruning rate to satisfy the time constraint at the same time.

## 5. Conclusions and Future Work

In this paper, we analyzed network compression methods (network simplification and parameter quantization) for real-time running DNN-based object detectors on edge devices through various experiments. In particular, five pruning methods considering the residual network structure were compared and it was found that the OR method is the best. In addition, it was found that when the detection difficulty of the dataset is low, the detection performance does not differ significantly depending on the parameter quantization method, but in other cases, QAT can prevent performance degradation. Finally, when the object is visually small, it was proven to be appropriate to increase the resolution of the input image to maintain the detection performance and to increase the pruning rate to reduce the inference time. The pruning methods compared in this paper do not consider how far the layer to be pruned is from the input and output nodes of the network. When the pruning rate of the layers close to the output node is high, it is expected that the detection result will change significantly before and after network pruning. Moreover, when the pruning rate of the layers close to the input node is high, it is expected that various primitive features will not be extracted. Therefore, in the future, we will study the pruning method considering the distance of each layer from the input and output node.

## Figures and Tables

**Table 1:** Description of VisDrone-DET2019.

Class	Pedestrian	People	Bicycle	Car	Van	Truck	Tricycle	Awning Tricycle	Bus	Motor
#GT(Train)	79,337	27,059	10,480	144,867	24,956	12,875	4812	3246	5926	29,647
#GT(Test)	8844	5125	1287	14,064	1975	750	1045	532	251	4886

**Table 2:** Description of SCOD.

Class	Vehicle	Pedestrian	Cyclist
#GT(Train)	49,131	49,794	6553
#GT(Test)	8563	5853	1378

**Table 3:** Comparison of detection performance according to quantization methods.

Dataset	Quantization Method	mAP (%)
VisDrone-DET2019	None (FP32)	19.68
	PTQ (INT8)	14.97
	QAT (INT8)	19.09
SCOD	None (FP32)	88.22
	PTQ (INT8)	89.48
	QAT (INT8)	88.73

**Table 4:** Detection performance of networks according to penalty coefficient α.

Dataset	Penalty Factor	mAP (%)
VisDrone-DET2019	0	19.68
	10^−3^	19.97
	10^−2^	19.73
	10^−1^	18.66
SCOD	0	87.99
	10^−4^	88.12
	10^−3^	87.23
	10^−2^	84.78

**Table 5:** Comparison pruning results in VisDrone-DET2019.

Pruning Rate	Method	mAP (%)	Parameter	BFLOPs	Inference Time (ms)		Volume (MB)
					GPU	DSP	
0	-	19.68	63.9 M	59.76	25	191	245.1
50	SK	20.35	19.5 M	38.03	24	128	74.8
	HF	20.39	17.5 M	35.27	23	123	67.1
	OR	20.65	19.1 M	37.89	23	127	73.2
	CC	19.99	19.4 M	39.96	24	139	74.5
	GA	19.99	18.2 M	36.36	24	260	69.8
70	SK	19.01	7.2 M	26.97	23	102	28.0
	HF	17.97	5.7 M	21.50	22	89	22.0
	OR	19.09	7.1 M	26.91	22	101	27.6
	CC	18.68	7.2 M	27.83	24	111	27.8
	GA	18.69	6.5 M	25.10	24	221	25.1

**Table 6:** Comparison pruning results in SCOD.

Pruning Rate	Method	mAP (%)	Parameter	BFLOPs	Inference Time (ms)		Volume (MB)
					GPU	DSP	
0	-	87.99	63.9 M	36.78	23	128	244.2
50	SK	87.87	16.6 M	24.26	22	81	63.9
	HF	88.45	15.9 M	23.71	22	81	61.1
	OR	89.09	16.4 M	24.21	21	80	63.1
	CC	88.22	16.8 M	25.74	23	88	64.7
	GA	88.22	15.6 M	23.47	22	196	61.8
70	SK	84.78	7.7 M	17.81	22	66	29.8
	HF	86.39	6.6 M	16.28	21	64	25.5
	OR	87.63	7.6 M	17.78	21	66	29.5
	CC	85.65	7.6 M	18.55	23	71	29.1
	GA	85.65	6.8 M	16.71	22	189	26.6

**Table 7:** Detection performance according to input image resolution and pruning ratio.

Dataset	Input Size	Prune Rate (%)	mAP (%)	Inference Time (ms)	Volume (MB)
VisDrone	384×384	60	18.24	94	46.6
	416×416	70	18.90	101	27.6
	448×448	80	18.84	98	13.6
SCOD	480×320	60	90.43	93	46.1
	512×352	70	90.64	99	28.8
	544×384	80	90.98	97	13.9

## Data Availability

Not applicable.
